# Impact of Chronic Obstruction Pulmonary Disease on Survival in Patients with Advanced Stage Lung Squamous Cell Carcinoma Undergoing Concurrent Chemoradiotherapy

**DOI:** 10.3390/cancers13133231

**Published:** 2021-06-28

**Authors:** Kuo-Chin Chiu, Wei-Chun Lin, Chia-Lun Chang, Szu-Yuan Wu

**Affiliations:** 1 Division of Chest, Department of Internal Medicine, Lo-Hsu Medical Foundation, Lotung Poh-Ai Hospital, Yilan 256, Taiwan; 979010@mail.pohai.org.tw (K.-C.C.); 973002@mail.pohai.org.tw (W.-C.L.); 2 Department of Hemato-Oncology, Wan Fang Hospital, Taipei Medical University, Taipei 110, Taiwan; richardch9@tmu.edu.tw; 3 Department of Internal Medicine, School of Medicine, College of Medicine, Taipei Medical University, Taipei 110, Taiwan; 4 Department of Food Nutrition and Health Biotechnology, College of Medical and Health Science, Asia University, Taichung 413, Taiwan; 5 Big Data Center, Lo-Hsu Medical Foundation, Lotung Poh-Ai Hospital, Yilan 256, Taiwan; 6 Division of Radiation Oncology, Lo-Hsu Medical Foundation, Lotung Poh-Ai Hospital, Yilan 256, Taiwan; 7 Department of Healthcare Administration, College of Medical and Health Science, Asia University, Taichung 413, Taiwan; 8 Cancer Center, Lo-Hsu Medical Foundation, Lotung Poh-Ai Hospital, Yilan 256, Taiwan; 9 Graduate Institute of Business Administration, Fu Jen Catholic University, Taipei 242062, Taiwan; 10 Centers for Regional Anesthesia and Pain Medicine, Taipei Municipal Wan Fang Hospital, Taipei Medical University, Taipei 110, Taiwan

**Keywords:** lung cancer, squamous cell carcinoma, chronic obstruction pulmonary disease, concurrent chemoradiotherapy, survival

## Abstract

**Simple Summary:**

No data are available regarding the effect of chronic obstruction pulmonary disease (COPD) and COPD with acute exacerbation (COPDAE) on survival in patients with lung squamous cell carcinoma (SCC) receiving definitive concurrent chemoradiotherapy (CCRT). This study is the first to examine the survival impact of COPD in patients with lung SCC receiving definitive CCRT. COPD and its severity are significant independent risk factors for all-cause mortality in patients with stage IIIA–IIIB lung SCC receiving definitive CCRT. Hospitalization for COPDAE within 1 year before CCRT is the significant independent risk factor for lung cancer death in the patients with stage IIIA–IIIB lung SCC receiving definitive CCRT.

**Abstract:**

Background: To date, no data are available regarding the effect of chronic obstruction pulmonary disease (COPD) and COPD with acute exacerbation (COPDAE) on survival in patients with lung squamous cell carcinoma (SCC) receiving definitive concurrent chemoradiotherapy (CCRT). Patients and methods: We enrolled 3986 patients with clinical stage IIIA–IIIB, unresectable lung SCC, who had received standard definitive CCRT, and categorized them into two groups based on their COPD status to compare overall survival outcomes. We also examined the effects of COPD severity (0, 1, or ≥2 hospitalizations for COPDA within 1 year before CCRT). Results: In the inverse probability of treatment weighting (IPTW)-adjusted model, the adjusted hazard ratio (aHR) (95% confidence interval (CI)) of all-cause death for COPD was 1.04 (1.01, 1.16), compared no COPD in patients with stage IIIA–IIIB lung SCC receiving definitive CCRT. In the IPTW-adjusted model, the aHRs (95% CIs) of 1 and ≥ 2 hospitalizations for COPDAE within 1 year before CCRT were 1.32 (1.19, 1.46) and 1.81 (1.49, 2.19) respectively, compared with no hospitalization for COPDAE. Conclusion: COPD and its severity are significant independent risk factors for all-cause death in patients with stage IIIA–IIIB lung SCC receiving definitive CCRT. Hospitalization for COPDAE within 1 year before CCRT is the significant independent risk factor for lung cancer death in the patients with stage IIIA–IIIB lung SCC receiving definitive CCRT.

## 1. Introduction

Chronic obstruction pulmonary disease (COPD) is an independent risk factor for lung carcinoma, particularly squamous cell carcinoma (SCC) [1]. COPD increases the risk of lung cancer by 6–13 times in the affected patients relative to individuals without COPD [2,3]. Lung cancer and COPD occur as comorbidities at a higher rate than would be expected if they were independently triggered [3]. Several mechanisms have been proposed to explain the association between COPD and lung cancer, including genetic risk factors [4], common epigenetic processes [5], and chronic local and systemic inflammatory processes [6]. In addition, lung cancer is the main cause of death among patients with COPD [7]. A detailed understanding of which clinical features of COPD increase the risk of lung cancer death is required [8]. COPD is associated with chronic local and systemic inflammatory processes in lung tissue [6], which might contribute to a low tolerance to curative-intent treatments, including radiotherapy (RT), lung lobectomy, or concurrent chemoradiotherapy (CCRT). Nevertheless, no data are available regarding the survival effects of non-COPD, COPD, or COPD with acute exacerbation (COPDAE) in patients with lung cancer across curative-intent treatments, pathologic type of lung cancer, or lung cancer stage. COPDAE contributes to long-term decline in lung function of patients with moderate to severe COPD [9]. COPDAE might be a risk factor for the prognostic factor of survival in patients with lung cancer, although there has been no data to prove this.

For most patients with clinically evident mediastinal lymph node (N2) lung SCC (stage IIIA–IIIB according to the American Joint Committee on Cancer (AJCC), Eighth Edition), the treatment approach is definitive CCRT using platinum-based chemotherapy plus full-dose RT [10,11,12]. For N2 lung SCC, definitive CCRT is the category 1 treatment approach as per the National Comprehensive Cancer Network (NCCN) guidelines [10,11,12]. Some studies have shown that COPD is associated with the risk of radiation-induced lung injury (RILI) [13,14]. Among 80 patients with stage IIIA–IIIB non-small-cell lung cancer treated with cisplatin-based chemotherapy and RT, COPD was associated with an increased frequency of RILI, including radiation pneumonitis (RP), an increased risk of severe pneumonitis, and late lung fibrosis [15,16]. COPD or severe COPD (COPDAE) associated with acute and late RILI might reduce survival in patients with lung SCC receiving definitive CCRT.

Combination CCRT with molecular targeting and/or immunotherapy could improve benefits. Durvalumab, which prevents the binding of PD-L1 to PD-1, was recently evaluated following CCRT using platinum-based doublet against placebo, and the results were encouraging [17]. Although it might be a good time to look at the possible advantages of combining RT with molecular-targeted medicines and immunotherapies to improve cancer survival in COPD patients, there were no EGFR mutations in our patients with advanced stage lung SCC. Thus, there is no indication for using molecular targeting as the first-line treatment for these patients with advanced stage lung SCC, only definitive CCRT to advanced stage lung SCC based on NCCN guidelines [12]. Moreover, Taiwan’s National Health Insurance payment for immunotherapy only started on 1 January 2019. However, we enrolled patients who had received a diagnosis of lung SCC with positive mediastinal lymph node (clinical N2 stage, AJCC stage IIIA–IIIB) between 1 January 2008 and 31 December 2017. Therefore, our database did not include the immunotherapy and there were no sufficient follow-up times to estimate the survival impact of COPD and severity of COPD for patents with lung SCC receiving CCRT followed by immunotherapy. In addition, the sample size, homogeneous treatments, and the same lung cancer type should be necessarily accumulated for longer follow-up time and larger sample size to estimate the survival impact on COPD and lung cancer with molecular targeting and/or immunotherapy. Therefore, we will design a new study in the near future to analyze our data and bring in some more granular information regarding the lessons learned from these trials to improve treatment options. Until now, no study has investigated the survival effects of non-COPD, COPD, and COPDAE in patients with lung SCC receiving definitive CCRT. In the current study, we examined whether COPD or COPDAE is a risk factor for all-cause death, COPD death, and lung cancer death in patients with stage IIIA–IIIB lung SCC receiving standard CCRT.

## 2. Patients and Methods

### 2.1. Study Population

In this cohort study, data were retrieved from the combination of two databases: the National Health Insurance Research Database (NHIRD) and the Taiwan Cancer Registry Database (TCRD). We enrolled current-smoking patients who had received a diagnosis of lung SCC with positive mediastinal lymph node (clinical N2 stage, AJCC stage IIIA–IIIB) between 1 January 2008 and 31 December 2017. The index date was the start date of definitive CCRT, and the follow-up duration was from the index date to 31 December 2018. The TCRD of the Collaboration Center of Health Information Application contains detailed cancer-related information of patients, including the clinical stage, treatment modalities, chemotherapy regimens, dose of chemotherapy, pathology, radiation modalities and doses, and treatment protocols (CCRT or non-CCRT) [18,19,20,21,22,23]. The study protocols were reviewed and approved by the Institutional Review Board of Tzu-Chi Medical Foundation (IRB109-015-B).

### 2.2. Inclusion and Exclusion Criteria

The diagnoses of the enrolled current-smoking patients were confirmed through a review of their pathological data, and patients with newly diagnosed lung SCC were confirmed to have no other cancers or distant metastases. Patients were included if they had received a lung SCC diagnosis, were aged ≥20 years, and had AJCC clinical stages IIIA–IIIB without metastasis. Patients were excluded if they had a history of cancer before lung SCC diagnosis, had distant metastasis, had unknown pathologic type, had missing sex data, were aged < 20 years, had unclear staging, or exhibited non-SCC histology. In addition, we excluded patients with lung SCC if they did not receive any treatments within 3 months after diagnosis, received an insufficient CCRT dose (< 6000 cGy) after lung SCC diagnosis, received insufficient chemotherapy (concurrent chemotherapy with two agents containing platinum at least), or did not receive a platinum-based chemotherapy regimen. We also excluded those who received only sequential chemotherapy and RT, chemotherapy alone, or RT alone. Standard CCRT comprises concurrent chemotherapy with two agents containing platinum and thoracic RT with 6000 cGy in daily fractions [24,25,26]. Finally, we enrolled 3986 patients with AJCC stages IIIA–IIIB unresectable lung SCC who had received definitive CCRT and assigned them into two groups based on their COPD status to compare overall survival outcomes: group 1 (patients with COPD before CCRT) and group 2 (patients without COPD before CCRT). We also investigated the effects of COPD severity (0, 1, or ≥ 2 hospitalizations for COPDAE within 1 year before the index date) on the survival outcomes in patients with stages IIIA–IIIB lung SCC receiving definitive CCRT. In the current study, we retrieved data for all patients with diagnoses of COPD from Taiwan’s NHIRD. The diagnoses of COPD were validated by the analysis of selected samples from the claims database of Taipei Veterans General Hospital (a 3035-bed tertiary referral hospital in Taiwan) using the same criteria as in the previous study [27]. Almost all medical services provided by Taipei Veterans General Hospital are covered and recorded in the NHIRD [27]. The enrolled patients were all current-smoking patients. However, the smoking intensity is not listed in Table 1 and Table 2, because of the strong collinearity of smoking intensity, COPDAE, histological degree of differentiation of the cancer, and smoking-associated complications. In statistics, multicollinearity (also collinearity) is a phenomenon in which one predictor variable in a multiple regression model can be linearly predicted from the others with a substantial degree of accuracy [28]. In this situation, the coefficient estimates of the multiple regression may change erratically in response to small changes in the model or the data [28]. The incidence of comorbidities was scored using the Charlson comorbidity index (CCI) [29,30]. Only the comorbidities observed in the 6 months before the index date were included, and they were coded and classified according to the International Classification of Diseases, 10th Revision, Clinical Modification (ICD-10-CM), at the first admission or after more than two repetitions of a code were issued at outpatient department visits. Supplementary Figure S1 shows the flow-chart of patient selection.

### 2.3. Study Covariates and Statistical Analysis

Significant independent predictors, namely age, sex, cancer stages, histological degree of differentiation, diabetes, chronic bronchitis, asthma, emphysema, cardiovascular disease (CVD), acute myocardial infarction (AMI), stroke, CCI score, income level, and urbanization, were analyzed using the multivariate Cox proportional hazard model to determine hazard ratios (HRs). We applied inverse probability of treatment weighting (IPTW) to create a pseudo-study cohort. The weighted cohort avoids covariate bias and mimics randomized COPD or non-COPD assignment: IPTW for patients with COPD = 1/p(COPD), and IPTW for patients without COPD = 1/(1 − p(COPD)) [31,32]. The independent predictors were examined in univariate and multivariable analyses before and after IPTW adjustment. The independent predictors were controlled for and were stratified in the analysis. The primary endpoint was the mortality rate in the patients with COPD, with group 1 used as the control. According to the cause of death profiles in NHIRD, we also supplied the secondary endpoints of death of COPD and death of cancer in Supplementary Tables S1 and S2.

The cumulative incidence of death was estimated using the Kaplan-Meier method, and differences in the frequency of hospitalization between patients with COPDAE, with COPD, and without COPD with lung SCC receiving definitive CCRT were determined using the log-rank test. After adjustment for confounders, IPTW-adjusted models were used to determine the time from the index date to all-cause mortality in the patients with COPD, with COPDAE, and without COPD. Subsequently, in multivariate analysis, HRs were adjusted for age, sex, cancer stages, histological degree of differentiation, diabetes, chronic bronchitis, asthma, emphysema, CVD, AMI, stroke, CCI score, income level, and urbanization. All analyses were conducted using SAS (Version 9.3; SAS, Cary, NC, USA), and a two-tailed *p*-value of < 0.05 was considered statistically significant.

## 3. Results

### 3.1. Study Cohort

We enrolled 3986 patients with stage IIIA–IIIB, unresectable lung SCC, who had received standard definitive CCRT and did not have distant metastases (Table 1). Among these patients, 1219 had COPD before definitive CCRT (group 1), and 2767 did not have COPD before definitive CCRT (group 2). The dosage distributions of radiation and chemotherapy between groups 1 and 2 were homogenous, and the median irradiation dose in both groups was 6300 cGy. The median follow-up durations after the index date were 1.96 and 1.09 years for patients without and with COPD, respectively. The two groups differed significantly in age, follow-up duration, sex, CCI score, diabetes, asthma, emphysema, CVD, AMI, stroke, income level, and urbanization (Table 1). More patients in the COPD group were old and male, had diabetes, chronic bronchitis, asthma, emphysema, CVD, AMI, stroke, CCI ≥ 2, low income, and lived in rural regions, compared to in the non-COPD group. However, the proportion of clinical stages IIIA and IIIB and histological degree of differentiation were balanced between groups 1 and 2. The mortality was 68.12% and 76.95% in the non-COPD and COPD groups, respectively. The lung cancer death was 64.69% and 73.10% in the non-COPD and COPD groups, respectively.

### 3.2. Effects of COPD and Hospitalization for COPDAE on Survival Outcomes of Patients with Lung SCC Receiving Definitive CCRT

IPTW-adjusted models indicated that COPD and the frequency of hospitalization for COPDAE were significant and poor independent predictors in the patients with lung SCC who had received definitive CCRT (Table 2). In the IPTW-adjusted model, the aHR (95% CI) of all-cause death for COPD was 1.06 (1.07, 1.71) (*p* = 0.0311; Table 2) compared with the non-COPD group. Moreover, in the IPTW-adjusted model, the aHRs (95% CIs) of 1 and ≥2 hospitalizations for COPDAE within 1 year before CCRT were 1.29 (1.16, 1.43; *p* < 0.0001) and 1.77 (1.41, 2.13; *p* < 0.0001) respectively, compared with no hospitalization for COPDAE (Table 2). In the IPTW-adjusted model, the aHRs (95% CIs) of COPD death for 1 and ≥2 hospitalizations for COPDAE within 1 year before CCRT were 1.45 (1.31, 2.61; *p* < 0.0001) and 2.03 (1.64, 3.41; *p* < 0.0001) respectively, compared with no hospitalization for COPDAE (Supplementary Table S1). In Supplementary Table S2, after the IPTW-adjusted model, the aHR (95% CI) of lung cancer death for COPD was 1.01 (0.67, 1.54) (*p* = 0.4117) compared with the non-COPD group. Additionally, in the IPTW-adjusted model, the aHRs (95% CIs) of 1 and ≥2 hospitalizations for COPDAE within 1 year before CCRT were 1.21 (1.09, 1.39; *p* < 0.0001) and 1.63 (1.34, 1.97; *p* < 0.0001) respectively, compared with no hospitalization for COPDAE (Supplementary Table S2).

### 3.3. Other Independent Predictors of Overall Survival in Patients with Lung SCC Receiving Definitive CCRT

Old age (>65 years), male sex, advanced stage (IIIB), moderate to high differentiation, CCI ≥ 2, low income, and residence in a rural region were identified as crucial independent predictors of overall survival (Table 2). After IPTW-adjustment for age, sex, cancer stages, differentiation, diabetes, chronic bronchitis, asthma, emphysema, CVD, AMI, stroke, CCI score, income level, and urbanization, the aHRs (95% CIs) of overall mortality for age ≤ 65 years, 65 years < age ≤ 75 years, and 75 years < age ≤ 85 years were 0.47 (0.43, 0.57), 0.51 (0.39, 0.66), and 0.73 (0.69, 0.82) respectively, compared with age > 85 years, and those of male sex, stage IIIB, moderate grade of differentiation, high grade of differentiation, CCI ≥ 2, income > NTD30,000, and residence in urban regions were 1.21 (1.10, 1.29), 1.20 (1.09, 1.41), 1.17 (1.57, 1.89), 1.35 (1.08, 1.85), 1.18 (1.07, 1.27), 0.61 (0.53, 0.77), and 0.79 (0.74, 0.87) compared with female sex, stage IIIA, low grade of differentiation, CCI = 0, income < NTD18,000, and residence in rural regions, respectively (Table 2).

### 3.4. Survival Curves of COPD and Hospitalization for COPDAE in Patients with Lung SCC Receiving Definitive CCRT

**Figure 1:**
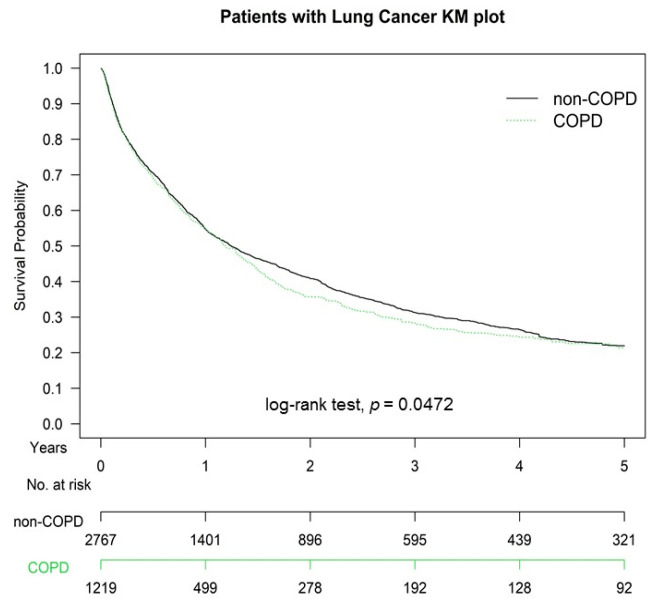
Kaplan-Meier overall survival curves of propensity score-weighted population of advanced stage lung squamous cell carcinoma with or without chronic obstruction pulmonary disease before concurrent chemoradiotherapy.

**Figure 2:**
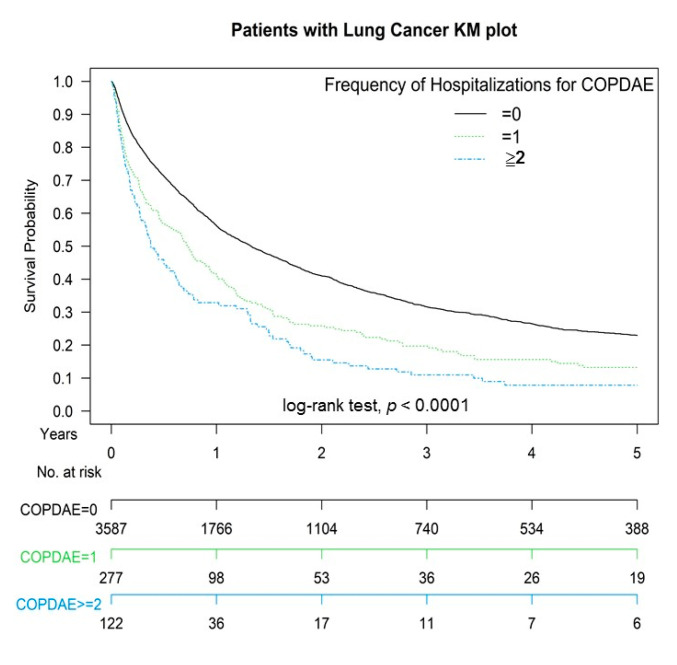
Kaplan-Meier overall survival curves of propensity score-weighted population of advanced stage lung squamous cell carcinoma according to the frequency of hospitalization for chronic obstruction pulmonary disease with acute exacerbation within 1 year before concurrent chemoradiotherapy.

Figure 1 presents Kaplan-Meier overall survival curves of propensity score-weighted population of advanced stage lung SCC with or without COPD before CCRT. Specifically, the two-year overall survival rates across all clinical stages were 32.38% and 22.81% in the non-COPD and COPD groups respectively (Figure 1), and the overall survival rate was higher in the non-COPD group (log-rank test, *p* = 0.0472) than in the COPD group. Additionally, the two-year overall survival rates in patients with lung SCC who were hospitalized for COPDAE 0, 1, and ≥2 times within 1 year before CCRT were 30.78%, 19.13%, and 17.93%, respectively (Figure 2; log-rank test, *p* < 0.0001).

## 4. Discussion

COPD and its severity (the frequency of hospitalization for COPDAE) might play an important role in acute or late radiation-induced lung toxicity [13,14,15,16]. In lung cancer, the addition of chemotherapy to RT might be more toxic for irradiated normal lung tissue due to the risks of RILI [33,34,35,36,37,38,39]. Based on the NCCN guidelines, definitive CCRT is considered a category 1 curative-intent treatment for N2 nodal-positive lung cancer [12]. Owing to the different preferred regimens of squamous and non-squamous cell carcinoma lung cancer based on the NCCN guidelines [10,11,12], we only enrolled patients with stage IIIA–IIIB (clinical N2-positive) lung SCC to keep the dose and regimens of CCRT consistent. In our design of consistent clinic stages and pathologic type, the effect of COPD or COPDAE on survival outcomes was clarified in the patients with stage IIIA–IIIB lung SCC receiving definitive CCRT, with similar chemotherapy and irradiation dose and volume. This study is the first to investigate the effects of COPD and COPDAE on the survival outcomes of patients with N2 stage lung SCC receiving definitive CCRT. Our study showed that COPDAE were not only a poor prognostic factor of all-cause death, but also a strongly poor prognostic factor of COPD death and lung cancer death for advanced lung SCC receiving definitive CCRT (Supplementary Tables S1 and S2). However, the preexisting COPD was the significant prognostic factor of all-cause death, instead of lung cancer death, in the patients with advanced lung SCC receiving definitive CCRT.

There are several small sample size, retrospective, inhomogeneous lung cancer types, different stages, and inconsistent treatment studies for the survival impact of COPD in treatments of lung cancers [40,41,42,43]. In the previous study, COPD is a risk factor for postoperative recurrence in non-small-cell lung cancer (NSCLC) patients, and moderate to severe COPD is an independent adverse prognostic factor for recurrence-free survival, which is very different from our endpoint (overall survival) [42]. A recent paper reported that coexisting COPD has an effect on the survival of patients with small-cell lung cancer who are undergoing chemotherapy, but different from ours who had lung squamous cell carcinoma (SCC) and were receiving standard CCRT [40]. Lim et al. have studied the impact of COPD status and systemic inflammation on the outcome of advanced NSCLC with a multicenter retrospective cohort study [41]. Lim et al.’s study showed a high platelet–lymphocyte ratio for the COPD group, who had a significantly higher risk for mortality compared with the low-platelet–lymphocyte ratio in the non-COPD group [41]. There were no consistent treatments for advanced NSCLC in Lim’s study [41]. In fact, there were different regimens of chemotherapy combined with RT for squamous cell carcinoma or adenocarcinoma according to NCCN guidelines [12]. There were no consistent pathologic types of lung cancer in the aforementioned studies [40,41,42]. Moreover, Zhai et al. studied the impact of coexisting COPD on the survival of patients with early-stage NSCLC undergoing surgical resection, instead of CCRT for advanced lung SCC, for which the impact of coexisting COPD for the surgery-related toxicity or complications might be different from our study of CCRT (definitive CCRT for clinical stage IIIA–IIIB lung SCC) [43]. Taken together, this study is the first to show that the frequency of hospitalization for COPDAE within 1 year before CCRT (similar to the Global Initiative for Chronic Obstructive Lung Disease (GOLD) Classification 3–4) [7] is a significant risk factor for all-cause death and lung cancer death for the patients with lung SCC receiving definitive CCRT.

As shown in Table 1, more patients in the COPD group were male and old, had diabetes, chronic bronchitis, asthma, emphysema, CVD, AMI, stroke CCI ≥ 2, and low income, and resided in rural regions, compared to in the non-COPD group. The distribution of patient characteristics was reasonable in this study and is compatible with that in previous studies, because patients with COPD have more comorbidities [7,44,45,46,47]. Although matching creates a balanced dataset by making pairs between controls and treated patients on the basis of a similar propensity score, some patients may be excluded from the dataset, which is a major disadvantage [48,49]. In the current study, IPTW (Table 2) was conducted, which has advantages over matching of patients based on propensity scores when there are two groups to compare, when finding matches results in insufficient sample sizes, or when the data are censored [48,49]. Moreover, the data in Table 1 are real-world data; thus, we did not use propensity score matching to wash out too much of a sample of patients, which induced a deviation database [48,49]. To create a pseudo-study cohort, where the weighted version can avoid the covariate bias and mimic randomized COPD or non-COPD assignment, we used IPTW-adjustment to identify the independent predictors of all-cause death in patients with lung SCC receiving CCRT [31,32]. In addition, we used the multivariate IPTW-adjustment model to determine the HRs of all-cause death, COPD death, and lung cancer death in these patients for identifying the independent predictors of all-cause death.

In IPTW-adjusted models, the independent predictors of all-cause death or lung cancer death were similar in patients with lung SCC receiving CCRT (Table 2 and Supplementary Table S2). In multivariate analysis through IPTW-adjustment, the patients with COPD with stage IIIA–IIIB lung SCC receiving definitive CCRT had worse survival compared with those without COPD receiving the same curative treatments (Table 2). However, COPD was not a significant prognostic factor of lung cancer death in our study. This study is the first to show that, among patients with lung SCC receiving definitive CCRT, those with COPD had a high risk of all-cause death compared to those without COPD, instead of a risk factor of lung cancer death. Another contribution of our study is confirming that the severity of COPD is strongly associated with survival in the patients with lung SCC receiving CCRT. The frequency of hospitalization for COPDAE was significantly associated with worse survival in the patients with lung SCC receiving CCRT (Table 2 and Figure 2). According to our literature review, this study is the first to show that the frequency of hospitalization for COPDAE within 1 year before CCRT (similar to the Global Initiative for Chronic Obstructive Lung Disease (GOLD) Classification 3–4) [7] is a significant risk factor for all-cause death, COPD death, and lung cancer death for the patients with lung SCC receiving definitive CCRT. The potential mechanism might be severe COPD (GOLD 3–4) associated with severe lung inflammation before CCRT [6]. Severe lung inflammation may be induced by COPD, resulting in worse survival after CCRT. According to a previous study, COPD influences the risk of RP [15]. Therefore, COPD or COPDAE might be associated with worse survival in the patients with stage IIIA–IIIB lung SCC receiving definitive CCRT. In addition, previous studies have demonstrated chemotherapy exposure, type of anticancer drug, and chemotherapy timing as risk factors for RP [33,34,35,36,37,38,39]. Animal studies and clinical reports have indicated the concomitant administration of RT and chemotherapy as risk factors for RP [33,34,35,36,37,38,39,50]. RILI might be associated with worse survival and poor pulmonary function in COPD and COPDAE patients, which might also contribute to poor survival in patients with lung SCC receiving definitive CCRT [7]. Moreover, COPD or COPDAE related to poor pulmonary function and CCRT related to RILI might be additive or synergistic risk factors for all-cause death in patients with lung SCC receiving CCRT. Our findings demonstrated that COPD and the frequency of hospitalization for COPDAE within 1 year before definitive CCRT were significant risk factors for overall survival in the patients with stage IIIA–IIIB lung SCC. For lung cancer death, COPD with relatively better pulmonary function compared with COPDAE was contributed to better tolerance of RILI induced by definitive CCRT. As a result, COPD is not the prognostic factor of lung cancer death in the patients with advanced lung SCC receiving definitive CCRT.

Very few studies have investigated the correlation between COPD, COPDAE, low income, and residence in rural regions and definitive CCRT in patients with stage IIIA–IIIB lung SCC. However, some small, retrospective studies have shown similar outcomes compatible with ours, showing that old age, male sex, moderate to high grade of differentiation, and high CCI scores are independent risk factors for all-cause death for lung cancer with nonhomogeneous stages, pathologic types, or various treatments [51,52,53]. Nevertheless, few studies have shown that COPD, COPDAE, income level, and urbanization are associated with the survival outcomes of patients with stage IIIA–IIIB lung SCC receiving CCRT. Our study revealed that COPDAE before CCRT are important risk factors for all-cause death and demonstrated that moderate to high grade of differentiation, old age, male sex, low income, and residence in rural regions are negative independent predictors of all-cause death and lung cancer death in the patients with lung SCC receiving CCRT (Table 2 and Supplementary Table S2).

According to our literature review, no peer-reviewed and large cohort study has investigated patients with COPD, COPDAE, and IIIA–IIIB lung SCC receiving standard CCRT regimens. This is the first study to examine the effects of COPD and COPDAE on the survival outcomes of patients with stage IIIA–IIIB lung SCC receiving definitive CCRT. In the future, COPD or hospitalization for COPDAE before definitive CCRT should be considered as valuable predictors in patients with stage IIIA–IIIB lung SCC receiving definitive CCRT. COPD and COPDAE are simple, easily measurable, and valuable predictors of all-cause death in patients with stage IIIA–IIIB lung SCC receiving CCRT, and they would enable shared decision-making between physicians and patients. In addition, although patients with COPD or COPDAE with stages IIIA–IIIB lung SCC receive definitive CCRT, radiation oncologists might consider more precision RT techniques, including image-guided radiation therapy, respirator gating, and other respiratory control techniques, for reducing RILI and ensuring low irradiation to normal lung, heart, and esophageal tissues to prevent acute or late radiation-induced pulmonary, cardiac, and esophageal toxicities in these patients [54,55].

This study has some limitations. First, toxicity induced by different treatments could not be determined; therefore, treatment-related mortality estimates may have been biased. Second, because all the patients with lung SCC were enrolled from an Asian population, the corresponding ethnic susceptibility remains unclear; hence, our results should be extrapolated to non-Asian populations with caution. Third, diagnoses of all comorbidities were completely dependent on ICD-10-CM codes. Nevertheless, the Taiwan Cancer Registry Administration randomly reviews charts and interviews patients to verify the accuracy of the diagnoses, and hospitals with outlier chargers or practices may undergo an audit and subsequently receive heavy penalties if malpractice or discrepancies are identified. Therefore, for obtaining crucial information concerning population specificity and disease occurrence, a large-scale randomized trial comparing carefully selected patients undergoing suitable treatments is essential. Finally, TCRD does not contain information regarding dietary habits or body mass index, and these factors may be risk factors for mortality. However, considering the magnitude and statistical significance of the observed effects in this study, these limitations are unlikely to affect the conclusions.

## 5. Conclusions

COPD and the frequency of hospitalization for COPDAE within 1 year before CCRT are significant independent risk factors for overall survival in the patients with stage IIIA–IIIB lung SCC receiving definitive CCRT. Hospitalization for COPDAE within 1 year before CCRT is the significant independent risk factor for lung cancer death in the patients with stage IIIA–IIIB lung SCC receiving definitive CCRT.

## Figures and Tables

**Table 1:** Characteristics of patients with advanced stage lung squamous cell carcinoma with or without chronic obstructive pulmonary disease before concurrent chemoradiotherapy.

	No COPD before CCRT *N* = 2767		COPD before CCRT *N* = 1219		*p*
	*N*	(%)	*N*	(%)	
Age (mean ± SD)	(65.42 ± 12.90)		(73.10 ± 10.25)		<0.0001
Age					<0.0001
Age ≤ 65 years	1330	48.07%	246	20.18%	
>65 years Age ≤ 75 years	743	26.85%	390	31.99%	
>75 years Age ≤ 85 years	557	20.13%	465	38.15%	
Age > 85 years	137	4.95%	118	9.68%	
Sex					<0.0001
Female	1233	44.56%	243	19.93%	
Male	1534	55.44%	976	80.07%	
AJCC clinical stage					0.9131
Stage IIIA	111	40.33%	499	40.94%	
Stage IIIB	1651	59.67%	720	59.06%	
Histological degree of differentiation					0.9137
Low	636	22.99%	292	23.95%	
Moderate	1165	42.10%	524	42.99%	
Diabetes					0.0041
No	2155	77.88%	898	73.67%	
Yes	612	22.12%	321	26.33%	
Chronic bronchitis					<0.0001
No	2705	97.76%	1101	90.32%	
Yes	62	2.24%	118	9.68%	
Asthma					<0.0001
No	2477	89.52%	717	58.82%	
Yes	290	10.48%	502	41.18%	
Emphysema					0.3231
No	2734	98.81%	1199	98.36%	
Yes	33	1.19%	20	1.64%	
Cardiovascular diseases					<0.0001
No	1119	40.44%	264	21.66%	
Yes	1648	59.56%	955	78.34%	
AMI					<0.0001
No	2703	97.69%	1144	93.85%	
Yes	64	2.31%	75	6.15%	
Stroke					<0.0001
No	2624	94.83%	1118	91.71%	
Yes	143	5.17%	101	8.29%	
CCI score					<0.0001
0	2330	84.21%	844	69.24%	
1	71	2.57%	49	4.02%	
≥2	366	13.23%	326	26.74%	
Income level					<0.0001
<NTD18,000	70	2.53%	58	4.76%	
NTD18,000–22,500	1538	55.58%	769	63.08%	
NTD22,500–30,000	726	26.24%	328	26.91%	
>NTD30,000	433	15.65%	64	5.25%	
Urbanization					<0.0001
Rural	815	29.45%	437	35.85%	
Urban	1952	70.55%	782	64.15%	
Hospitalizations for COPDAE within 1 year before diagnosis					<0.0001
0	2767	100.00%	820	67.27%	
1	0	0.00%	277	22.72%	
≥2	0	0.00%	122	10.01%	
Follow-up years Median (IQR, Q1, Q3)	1.92 (0.34, 2.63)		1.09 (0.22, 1.78)		<0.0001
All-cause death	1885 68.12%		938 76.95%		<0.0001
COPD death	0 0%		36 2.95%		<0.0001
Lung cancer death	1790 64.69%		891 73.10%		<0.0001

COPD, chronic obstruction pulmonary disease; CCRT, concurrent chemoradiotherapy; COPDAE, COPD with acute exacerbation; AJCC, American Joint Committee on Cancer; CCI, Charlson comorbidity index; AMI, acute myocardial infarction; NTD, New Taiwan Dollar; SD, standard deviation; IQR, interquartile range.

**Table 2:** Multivariable Cox regression of all-cause death with propensity score inverse probability of treatment weighting for patients with advanced stage lung squamous cell carcinoma with or without chronic obstructive pulmonary disease before concurrent chemoradiotherapy.

	Crude HR (95% CI)		Adjusted HR (95% CI) *		*p*
COPD (ref. non-COPD)					
COPD	1.37	(1.26, 1.51)	1.06	(1.07, 1.71)	0.0311
Frequency of hospitalizations for COPDAE before diagnosis (ref. 0)					
1	1.51	(1.38, 1.74)	1.29	(1.16, 1.43)	<0.0001
≥2	1.97	(1.61, 2.29)	1.77	(1.41, 2.13)	<0.0001
Age (ref. Age > 85 years)					
Age ≤ 65 years	0.38	(0.28, 0.43)	0.47	(0.43, 0.57)	<0.0001
>65 years Age ≤ 75 years	0.45	(0.39, 0.51)	0.51	(0.39, 0.66)	<0.0001
>75 years Age ≤ 85 years	0.67	(0.59, 0.79)	0.73	(0.69, 0.82)	<0.0001
Sex (ref. Female)					
Male	1.47	(1.39, 1.63)	1.21	(1.10, 1.29)	<0.0001
AJCC clinical stage (ref. Stage IIIA)					
Stage IIIB	1.28	(1.06, 1.39)	1.20	(1.09, 1.41)	<0.0001
Histological degree of differentiation (ref. Low)					
Moderate	1.07	(0.72, 1.95)	1.17	(1.57, 1.89)	0.003
High	1.12	(0.63, 1.53)	1.35	(1.08, 1.85)	0.009
CCI score (ref. 0)					
1	1.42	(1.199, 1.74)	1.12	(0.81, 1.11)	0.3181
≥2	1.36	(1.22, 1.61)	1.18	(1.07, 1.27)	<0.0001
Diabetes (ref. No)					
Yes	1.08	(0.89, 1.29)	1.06	(0.84, 1.25)	0.3530
Chronic bronchitis (ref. No)					
Yes	1.03	(0.89, 1.07)	1.02	(0.83, 1.09)	0.1911
Asthma (ref. No)					
Yes	1.04	(0.87, 1.28)	1.04	(0.71, 1.24)	0.2719
Emphysema (ref. No)					
Yes	1.11	(0.95, 1.29)	1.08	(0.91, 1.29)	0.2619
Cardiovascular diseases (ref. No)					
Yes	1.07	(0.95, 1.18)	1.04	(0.87, 1.27)	0.5416
AMI (ref. No)					
	1.05	(0.90, 1.07)	1.03	(0.89, 1.13)	0.3801
Stroke (ref. No)					
	1.01	(0.91, 1.06)	1.00	(0.82, 1.09)	0.4797
Income level (ref. <NTD18,000)					
NTD18,000–22,500	0.93	(0.71, 1.19)	0.93	(0.81, 1.13)	0.2461
NTD22,500–30,000	0.81	(0.68, 0.97)	0.81	(0.71, 1.11)	0.0881
>NTD30,000	0.68	(0.41, 0.81)	0.61	(0.53, 0.77)	<0.0001
Urbanization (ref. Rural)					
Urban	0.80	(0.71, 0.88)	0.79	(0.74, 0.87)	<0.0001

* All covariates mentioned in Table 2 were adjusted. COPD, chronic obstruction pulmonary disease; CCRT, concurrent chemoradiotherapy; COPDAE, COPD with acute exacerbation; AJCC, American Joint Committee on Cancer; CCI, Charlson comorbidity index; AMI, acute myocardial infarction; NTD, New Taiwan Dollar; Ref, reference group; aHR, adjusted hazard ratio; CI, confidence interval; HR, hazard ratio.

## Data Availability

The data sets supporting the study conclusions are included in this manuscript and its supplementary files.

## Supplementary Materials

The following are available online at https://www.mdpi.com/article/10.3390/cancers13133231/s1, Figure S1: Flow-chart of patient selection, Table S1: Multivariable Cox regression of COPD death with propensity score inverse probability of treatment weighting for patients with advanced stage lung squamous cell carcinoma with or without chronic obstructive pulmonary disease before concurrent chemoradiotherapy, Table S2: Multivariable Cox regression of lung cancer death with propensity score inverse probability of treatment weighting for patients with advanced stage lung squamous cell carcinoma with or without chronic obstructive pulmonary disease before concurrent chemoradiotherapy.

## Abbreviations

COPD	chronic obstruction pulmonary disease
SCC	squamous cell carcinoma
CCRT	concurrent chemoradiotherapy
COPDAE	COPD with acute exacerbation
aHR	adjusted hazard ratio
CI	confidence interval
IPTW	inverse probability of treatment weighting
RT	radiotherapy
AJCC	American Joint Committee on Cancer
RILI	radiation-induced lung injury
RP	radiation pneumonitis
TCRD	Taiwan Cancer Registry Database
CCI	Charlson comorbidity index
ICD-10-CM	International Classification of Diseases, 10th Revision, Clinical Modification
CVD	cardiovascular disease
AMI	acute myocardial infarction
NTD	New Taiwan Dollar
NCCN	National Comprehensive Cancer Network
GOLD	Global Initiative for Chronic Obstructive Lung Disease
