# Supplementary material for: Impact of Chronic Obstruction Pulmonary Disease on Survival in Patients with Advanced Stage Lung Squamous Cell Carcinoma Undergoing Concurrent Chemoradiotherapy
Source: Cancers (Basel). 2021 Jun 28;13(13):3231. doi: 10.3390/cancers13133231 (PMC8268442; doi:10.3390/cancers13133231)
Supplement: Supplementary file 1 [file cancers-13-03231-s001.zip › cancers-1286599-supplementary.pdf]

cancers

MDPI

Supplementary Materials: Impact of Chronic Obstruction Pulmonary Disease on Survival in Patients with Advanced Stage Lung Squamous Cell Carcinoma Undergoing Concurrent Chemoradiotherapy

Supplemental Table S1. Multivariable Cox regression of COPD death with propensity score inverse probability of treatment weighting for patients with advanced stage lung squamous cell carcinoma with or without chronic obstructive pulmonary disease before concurrent chemoradiotherapy.

	Crude HR (95% CI)		Adjusted HR (95% CI)*		*p*
Frequency of hospitalizations for COPDAE before diagnosis (ref. 0)					
1	1.69	(1.48,2.94)	1.45	(1.31,2.61)	<0.0001
≥2	2.24	(1.82,3.67)	2.03	(1.64,3.41)	<0.0001
Age (ref. Age > 85 years)					
Age ≤ 65 years	0.31	(0.26,0.76)	0.54	(0.49,0.71)	<0.0001
65 years < Age ≤ 75 years	0.44	(0.37,0.71)	0.46	(0.42,0.71)	<0.0001
75 years < Age ≤ 85 years	0.67	(0.58,0.98)	0.81	(0.75,0.88)	<0.0001
Sex (ref. Female)					
Male	1.34	(1.23,3.45)	1.07	(1.02,3.13)	<0.0001
AJCC clinical stage (ref. Stage IIIA)					
Stage IIIB	0.88	(0.73,2.08)	0.95	(0.86,2.08)	0.2287
Histological degree of differentiation (ref. Low)					
Moderate	0.93	(0.65,2.75)	1.05	(0.92,2.51)	0.4523
High	1.08	(0.57,3.38)	0.89	(0.86,3.11)	0.3885
CCI score (ref. 0)					
1	1.30	(1.05,2.58)	0.83	(0.70,2.07)	0.3791
≥2	1.28	(1.17,3.57)	1.11	(1.05,2.18)	<0.0001
Diabetes (ref. No)					
Yes	1.07	(0.98,2.17)	1.03	(0.72,1.96)	0.3479
Chronic bronchitis (ref. No)					
Yes	1.25	(1.03,2.27)	1.22	(1.03,1.53)	0.0056
Asthma (ref. No)					
Yes	1.12	(1.03,1.51)	1.26	(1.11,318)	0.0078
Emphysema (ref. No)					
Yes	1.76	(1.61,1.93)	1.27	(1.14,1.78)	0.0051
Cardiovascular diseases (ref. No)					
Yes	1.49	(1.32,1.76)	1.31	(1.15,1.58)	0.0069
AMI (ref. No)					
	1.65	(1.10,2.19)	1.43	(1.07,1.75)	0.0012
Stroke (ref. No)					
	1.11	(0.81,1.97)	1.10	(0.91,1.42)	0.4205
Income level (ref. <NTD18,000)					
NTD18,000–22,500	0.92	(0.61,1.21)	1.04	(0.87,1.10)	0.0910
NTD22,500–30,000	0.81	(0.57,1.15)	0.77	(0.51,1.11)	0.1710
>NTD30,000	0.71	(0.41,0.89)	0.70	(0.47,0.85)	<0.0001
Urbanization (ref. Rural)					
Urban	0.77	(0.64,1.91)	0.91	(0.69,1.75)	0.8175

*All covariates mentioned in Table 3 were adjusted. COPD, chronic obstruction pulmonary disease; CCRT, concurrent chemoradiotherapy; COPDAE, COPD with acute exacerbation; AJCC, American Joint Committee on Cancer; CCI, Charlson comorbidity index; AMI, acute myocardial infarction; NTD, New Taiwan Dollar; Ref, reference group; aHR, adjusted hazard ratio; CI, confidence interval; HR, hazard ratio.

Supplemental Table S2. Multivariable Cox regression of lung cancer death with propensity score inverse probability of treatment weighting for patients with advanced stage lung squamous cell carcinoma with or without chronic obstructive pulmonary disease before concurrent chemoradiotherapy.

	**Crude HR (95% CI)**		**Adjusted HR (95% CI)***		***p***
COPD (ref. non-COPD)					
COPD	1.08	(0.77,1.66)	1.01	(0.67,1.54)	0.4117
Frequency of hospitalizations for COPDAE before diagnosis (ref. 0)					
1	1.36	(1.19,1.60)	1.21	(1.09,1.39)	<0.0001
≥2	1.81	(1.47,2.21)	1.63	(1.34,1.97)	<0.0001
Age (ref. Age > 85 years)					
Age ≤ 65 years	0.31	(0.25,0.36)	0.53	(0.49,0.61)	<0.0001
65 years < Age ≤ 75 years	0.48	(0.41,0.59)	0.52	(0.42,0.62)	<0.0001
75 years < Age ≤ 85 years	0.73	(0.61,0.85)	0.81	(0.75,0.88)	<0.0001
Sex (ref. Female)					
Male	1.34	(1.23,1.55)	1.07	(1.01,1.14)	<0.0297
AJCC clinical stage (ref. Stage IIIA)					
Stage IIIB	1.38	(1.16,1.54)	1.21	(1.18,1.50)	<0.0001
Histological degree of differentiation (ref. Low)					
Moderate	1.18	(1.05,2.11)	1.17	(1.23,2.09)	<0.0001
High	1.24	(1.16,1.78)	1.48	(1.20,2.04)	<0.0001
CCI score (ref. 0)					
1	1.12	(0.99,1.58)	1.01	(0.70,1.03)	0.4301
≥2	1.15	(1.04,1.28)	1.03	(0.96,1.09)	0.6719
Diabetes (ref. No)					
Yes	1.07	(0.91,1.17)	1.01	(0.73,1.16)	0.3117
Chronic bronchitis (ref. No)					
Yes	1.01	(0.78,1.07)	1.00	(0.73,1.07)	0.8619
Asthma (ref. No)					
Yes	0.91	(0.81,1.14)	0.95	(0.67,1.26)	0.4304
Emphysema (ref. No)					
Yes	1.14	(0.94,1.24)	0.97	(0.77,1.13)	0.3536
Cardiovascular diseases (ref. No)					
Yes	0.95	(0.88,1.05)	0.96	(0.79,1.11)	0.4988
AMI (ref. No)					
	0.93	(0.89,1.13)	0.91	(0.82,1.06)	0.1590
Stroke (ref. No)					
	0.97	(0.81,1.07)	0.94	(0.83,1.21)	0.4190
Income level (ref. <NTD18,000)					
NTD18,000–22,500	0.97	(0.66,1.21)	0.92	(0.89,1.20)	0.3426
NTD22,500–30,000	0.84	(0.68,1.03)	0.88	(0.70,1.04)	0.1967
>NTD30,000	0.46	(0.36,0.58)	0.70	(0.58,0.86)	<0.0001
Urbanization (ref. Rural)					
Urban	0.73	(0.62,0.80)	0.75	(0.71,0.81)	<0.0001

*All covariates mentioned in Table 3 were adjusted. COPD, chronic obstruction pulmonary disease; CCRT, concurrent chemoradiotherapy; COPDAE, COPD with acute exacerbation; AJCC, American Joint Committee on Cancer; CCI, Charlson comorbidity index; AMI, acute myocardial infarction; NTD, New Taiwan Dollar; Ref, reference group; aHR, adjusted hazard ratio; CI, confidence interval; HR, hazard ratio.

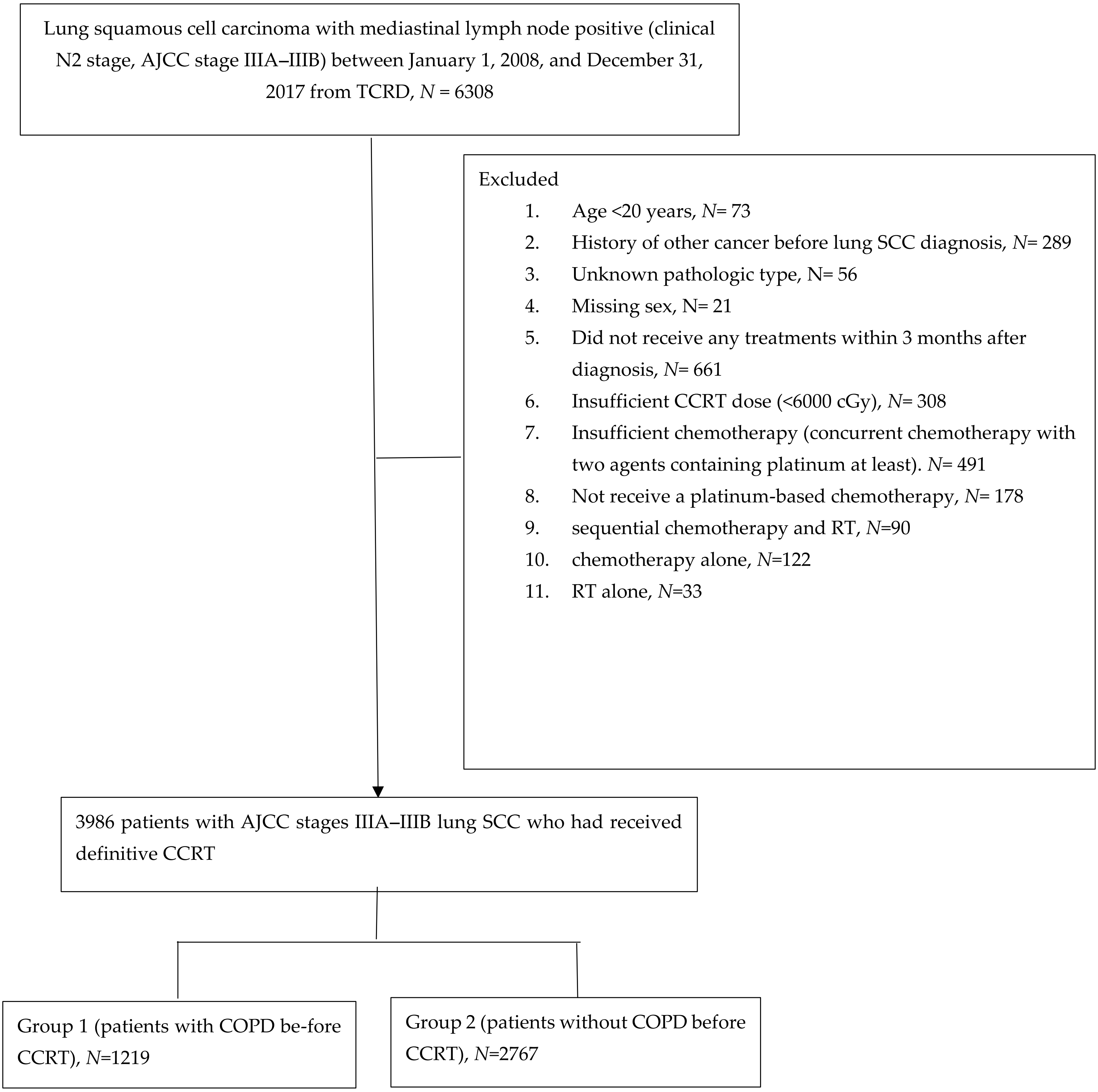

Supplemental Figure S1. Flow-chart of patient selection.
